# Correction to: Behavioral assay and chemical characters of female sex pheromones in the hermit crab *Pagurus filholi*

**DOI:** 10.1007/s10164-017-0535-7

**Published:** 2017-12-01

**Authors:** Saori Okamura, Takuma Kawaminami, Hiroshi Matsuura, Nobuhiro Fusetani, Seiji Goshima

**Affiliations:** 1 Laboratory of Marine Biology, Graduate School of Fisheries Sciences, Hokkaido University, Hakodate, Hokkaido 041-8611 Japan; 2 Division of Marine Life Science, Faculty of Fisheries Sciences, Hokkaido University, Hakodate, Hokkaido 041-8611 Japan; 3 Division of Marine Resource and Environmental Science, Faculty of Fisheries Sciences, Hokkaido University, Hakodate, Hokkaido 041-8611 Japan; 4 Present Address: Fisheries Science Center, Hokkaido University Museum, Hakodate, Hokkaido 041-8611 Japan

## Correction to: J Ethol (2017) 35:169–176 10.1007/s10164-017-0507-y

The article, “Behavioral assay and chemical characters of female sex pheromones in the hermit crab Pagurus filholi”, written by Saori Okamura, Takuma Kawaminami, Hiroshi Matsuura, Nobuhiro Fusetani, and Seiji Goshima was originally published Online First without open access. After publication in volume 35, issue 2, page 169-176 the author decided to opt for Open Choice and to make the article an open access publication. Therefore, the copyright of the article has been changed to © The Author(s) 2018 and the article is forthwith distributed under the terms of the Creative Commons Attribution 4.0 International License (http://creativecommons.org/licenses/by/4.0/), which permits use, duplication, adaptation, distribution and reproduction in any medium or format, as long as you give appropriate credit to the original author(s) and the source, provide a link to the Creative Commons license, and indicate if changes were made.

